# Supplementary material for: TBX5 genetic variants and SCD-CAD susceptibility: insights from Chinese Han cohorts
Source: PeerJ. 2024 Mar 19;12:e17139. doi: 10.7717/peerj.17139 (PMC10959103; doi:10.7717/peerj.17139)
Supplement: Supplemental Information 5 [file peerj-12-17139-s005.pdf]

GENETIC TESTING BIOTECHNOLOGY

Cell Line Authentication Service
STR Profile Report

Sample Submitted By: Dr. Yuzhen Gao
Soochow University
Email Address: yuzhengao@suda.edu.cn
Sales Order: 231108F
Cell Line Designation: HEK293T
Date Sample Received: Nov 8th, 2023
Report Date: Nov 8th, 2023

Methodology: Twenty-one short tandem repeat (STR) loci plus the Amelogenin locus were amplified using the commercially available SiFaSTR™ 23 plex Kit. The cell line sample was processed using the ABI Prism® 3130 XL Genetic Analyzer. Data were analyzed using GeneMapper® ID v3.2 software (Applied Biosystems). Appropriate positive and negative controls were run and confirmed for each sample submitted.

Data Interpretation: Cell lines were authenticated using Short Tandem Repeat (STR) analysis as described in 2021 in ANSI Standard (ASN-0002) by the ATCC Standards Development Organization (SDO) and in Jamie L. Almeida et al., Authentication of Human and Mouse Cell Lines by Short Tandem Repeat (STR) DNA Genotype Analysis. Assay Guidance Manual. PMID: 23805434. Bookshelf ID: NBK144066.

GTB™ performs STR Profiling following ISO 9001:2008 and ISO/IEC 17025:2005 quality standards.
There are no warranties with respect to the services or results supplied, express or implied, including, without limitation, any implied warranty of merchantability or fitness for a particular purpose. Genetic Testing Biotechnology (GTB) is not liable for any damages or injuries resulting from receipt and/or improper, inappropriate, negligent or other wrongful use of the test results supplied, and/or from misidentification, misrepresentation, or lack of accuracy of those results. Your exclusive remedy against GTB and those supplying materials used in the services for any losses or damage of any kind whatsoever, whether in contract, tort, or otherwise, shall be, at GTB's option, refund of the fee paid for such service or repeat of the service.

NOTE: According to the recommendations of *IJC* on cell line authentication, the report is valid for 3 years since the issue date.

Technical Questions?
GTB Technical Support
+86-512-67486171
service@jsdna.org
Section 505, Yixin BLD
SIP, Suzhou, 215123
Jiangsu, P.R. China

Ordering Questions?
order@jsdna.org
GTB Corporation
+86-512-62806339
Section 303, Yixin BLD
SIP, Suzhou, 215123
Jiangsu, P.R. China

GENETIC TESTING BIOTECHNOLOGY

Cell Line Authentication Service
STR Profile Report

Sales Order: 231108F

	Test Results for Submitted Sample		ExPASy Reference Database Profile	
Loci	Query Profile: HEK293T		Database Profile: HEK293T	
Amelogenin	X			
D3S1358	15	17	15	17
D5S818	8	9	8	9
D2S1338	19			
TPOX	11		11	
CSF1PO	11	12	11	12
Penta D	9	10		
TH01	7	9.3	7	9.3
vWA	16	19	16	19
D7S820	11		11	
D21S11	28	30.2	28	30.2
Penta E	7	15		
D10S1248	14			
D8S1179	12	14	12	14
D1S1656	15	17.3		
D18S51	17	18	17	18
D12S391	19	21		
D6S1043	11			
D19S433	18			
D16S539	9	13	9	13
D13S317	12	14	12	14
FGA	23		23	

The allele match algorithm compares the 13 core STR loci only, even though alleles from all loci will be reported when available.

Note: Loci highlighted in grey (13 core STR loci) can be made public to verify cell identity. In order to protect the identity of the donor, ***please do not publish*** *the allele calls from all the STR loci tested.*

The sample match is based on the reference data available at the time of comparison.

Explanation of Test Results

Cell lines with ≥80% match are derived from the same donor. Cell lines with between a 70% to 79% match require further profiling for authentication of relatedness. Cell lines with <70% match are very unlikely to be from the same donor.

- [ ] The submitted sample profile is human, but not a match for any profile in the ExPASy STR database.
- [x] The submitted profile is an exact match for the following human cell line(s) in the ExPASy STR database (13 core STR loci): HEK293T
- [ ] The submitted profile is similar to the following ExPASy human cell line(s):

e-Signature, Technician: Digitally signed by Xuekun Chen
DN: cn=Xuekun Chen, o=Genetic Testing Biotechnology (Suzhou), ou=DNA Typing Section, email=order@jsdna.org, c=CN
Date: 2023.11.08 11:14:05 +08'00'

e-Signature, Reviewer: Digitally signed by Xiankun Zhao
DN: cn=Xiankun Zhao, o=Genetic Testing Biotechnology (Suzhou), ou=Supervision Section, email=service@jsdna.org, c=CN
Date: 2023.11.08 11:14:43 +08'00'

GENETIC TESTING BIOTECHNOLOGY · GUARANTEE · CELL LINE AUTHENTICATION

More information

Addendum: Electropherogram for the customer's sample set 1 of 1

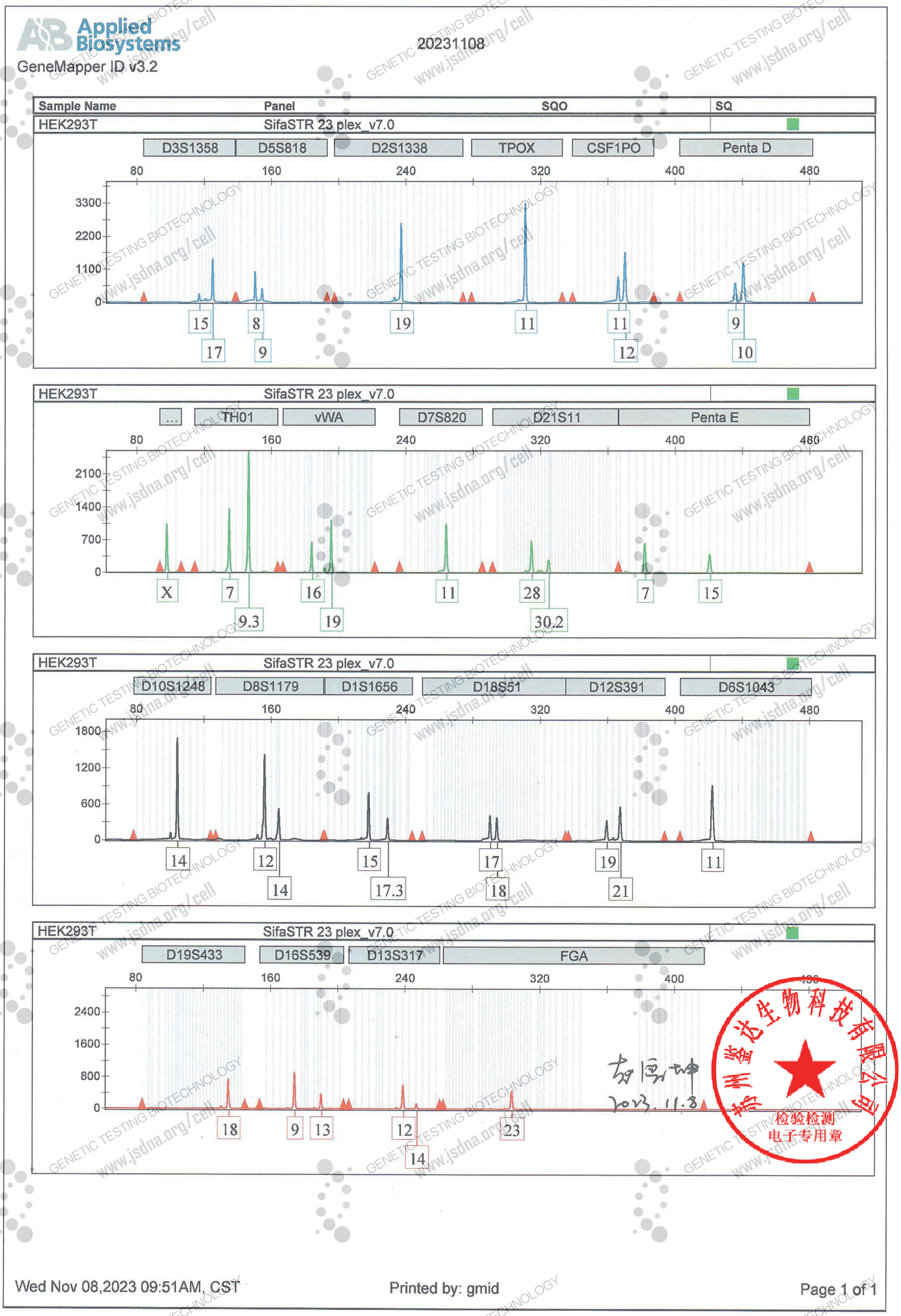
Applied Biosystems
GeneMapper ID v3.2
20231108
Sample Name
Panel
SQO
SQ
HEK293T
SifaSTR 23 plex_v7.0
D3S1358
D5S818
D2S1338
TPOX
CSF1PO
Penta D
15
17
8
9
19
11
11
12
9
10
HEK293T
SifaSTR 23 plex_v7.0
TH01
vWA
D7S820
D21S11
Penta E
X
7
9.3
16
19
11
28
30.2
7
15
HEK293T
SifaSTR 23 plex_v7.0
D10S1248
D8S1179
D1S1656
D18S51
D12S391
D6S1043
14
12
14
15
17.3
17
18
19
21
11
HEK293T
SifaSTR 23 plex_v7.0
D19S433
D16S539
D13S317
FGA
18
9
13
12
14
23
检验检测
电子专用章
Wed Nov 08,2023 09:51AM, CST
Printed by: gmid
Page 1 of 1
